# Novel methicillin resistance gene *mecD* in clinical *Macrococcus caseolyticus* strains from bovine and canine sources

**DOI:** 10.1038/srep43797

**Published:** 2017-03-08

**Authors:** Sybille Schwendener, Kerstin Cotting, Vincent Perreten

**Affiliations:** 1 Institute of Veterinary Bacteriology, Vetsuisse Faculty, University of Bern, Bern, Switzerland

## Abstract

Methicillin-resistant *Macrococcus caseolyticus* strains from bovine and canine origins were found to carry a novel *mecD* gene conferring resistance to all classes of β-lactams including anti-MRSA cephalosporins. Association of β-lactam resistance with *mecD* was demonstrated by gene expression in *S. aureus* and deletion of the *mecD*-containing island in *M. caseolyticus*. The *mecD* gene was located either on an 18,134-bp *M. caseolyticus* resistance island (McRI_*mecD*_-1) or a 16,188-bp McRI_*mecD*_-2. Both islands were integrated at the 3′ end of the *rpsI* gene, carried the *mecD* operon (*mecD-mecR1*_*m*_*-mecI*_*m*_), and genes for an integrase of the tyrosine recombinase family and a putative virulence-associated protein (*virE*). Apart from the *mecD* operon, that shared 66% overall nucleotide identity with the *mecB* operon, McRI_*mecD*_ islands were unrelated to any *mecB*-carrying elements or staphylococcal cassette chromosome *mec*. Only McRI_*mecD*_-1 that is delimitated at both ends by direct repeats was capable of circular excision. The recombined excision pattern suggests site-specific activity of the integrase and allowed identification of a putative core attachment site. Detection of *rpsI*-associated integrases in *Bacillus* and *S. aureus* reveals a potential for broad-host range dissemination of the novel methicillin resistance gene *mecD*.

*Macrococcus* is evolutionarily closely related to the genus *Staphylococcus*, but possesses a distinctly smaller genome with a size of 2.1 Mb in the species *M. caseolyticus*^1^. There are currently eight species included in the genus *Macrococcus*, namely *M. caseolyticus, M. equipercicus, M. bovicus, M. carouselicus, M. brunensis, M. hajekii, M. lamae* and *M. canis*^2^,^3^,^4^. *Macrococcus* is mainly found commensally on the surface of the body of animals and has also been isolated from raw milk and dairy products^5^,^6^,^7^. Methicillin-resistant strains were reported for *M. caseolyticus* from chicken, bovine milk and humans^1^,^8^,^9^,^10^ and for other *Macrococcus* species from dogs^11^,^12^.

Methicillin resistance in *Staphylococcus* and *Macrococcus* is due to the production of an alternative penicillin-binding protein (PBP2a, also called PBP2′ or MecA, MecB, and MecC) that has low affinity for almost all β-lactams^13^,^14^. In the presence of drug concentrations that inactivate native PBPs through irreversible acylation, PBP2a conserves its transpeptidase activity allowing cell-wall biosynthesis to continue^15^. PBP2a is encoded by a structural *mec* gene so far identified as *mecA, mecB* and *mecC*, and its presence can be predicted phenotypically using a screen for cefoxitin or oxacillin resistance^16^. The *mec* gene is organized in an operon with its regulators *mecR1* and *mecI* coding for an integral-membrane sensor/transducer and a transcriptional repressor, respectively^17^. The *mec*-*mecR1*-*mecI* gene complex is homologous to *blaZ*-*blaR1*-*blaI* controlling β-lactamase expression through an inducible proteolytic pathway^18^ and crosstalk between the systems has been observed^19^,^20^,^21^,^22^.

The classical *mecA* gene is carried by a unique class of mobile genetic elements, the staphylococcal cassette chromosome *mec* (SCC*mec*)^23^, originally found in methicillin-resistant *S. aureus* (MRSA)^24^. Since then, a wide structural diversity of SCC*mec* elements has been described in both coagulase-positive and coagulase-negative staphylococci that carry either the *mecA* or the *mecC* gene^25^. The SCC*mec* elements are site-specifically integrated into the chromosomal *orfX* gene, have characteristic flanking repeat sequences, a *mec* gene complex and cassette chromosome recombinase(s) (*ccr*) responsible for integration/excision of the element.

In *Macrococcus*, methicillin resistance has been associated with the *mecB* gene (formerly *mecA*_*m*_), a distantly related *mecA* homologue^1^. It is present in a *mec* gene complex (*blaZ*_*m*_*-mecB-mecR1*_*m*_*-mecI*_*m*_) which includes the regulators and additionally the *blaZ* homologue *blaZ*_*m*_. The *mecB* gene complex was originally found associated with transposon Tn*6045* located on either large plasmids or integrated into the chromosome as part of a SCC*mec*-like element in *M. caseolyticus*^1^,^8^. Recently, a SCC*mec* carrying *mecB* independent of Tn*6045* was reported in a hemolytic *Macrococcus* strain (*M. canis* sp. nov., formerly identified as *M. caseolyticus*)^4^,^12^.

Since 2014, several cases of bovine mastitis and an infection in a dog caused by methicillin-resistant *mecB*-negative *M. caseolyticus* strains were observed by our diagnostic unit at the Institute of Veterinary Bacteriology in Bern, Switzerland. This prompted us to investigate the nature of this β-lactam resistance by whole genome sequencing and gene expression which revealed the new methicillin resistance gene *mecD* on novel resistance islands.

## Results and Discussion

### Identification of a novel acquired PBP2a in *M. caseolyticus*

**Figure 1:**
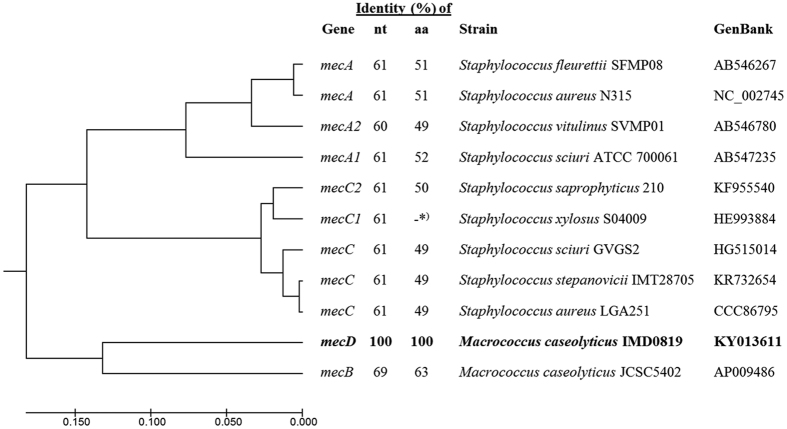
Phylogenetic tree of *mec* genes encoding PBP2a. Evolutionary analysis was performed for nucleotide sequences using the UPGMA method in MEGA7^56^. The percentage of nucleotide (nt) and amino acid (aa) identity between *mecD* and other *mec* genes was determined by sequence alignment with Clustral OMEGA [ http://www.ebi.ac.uk/Tools/msa/clustalo/]. *) *mecC1* of *S. xylosus* S04009 does not encode a functional PBP2a due to a frameshift mutation close to the 5′ end of the gene.

*M. caseolyticus* strains IMD0819 and IMD0473 were isolated from bovine mastitis milk and strain KM0211 from an infection site of a dog (Table 1). They all exhibited resistance to penicillin, cefoxitin and oxacillin but did not carry any of the described methicillin-resistance genes (Table 1). Whole genome sequencing and tblastn analysis using PBP2a encoded by *mecA, mecB* and *mecC* as queries revealed a novel *mec* gene homologue in the three β-lactam-resistant strains but not in the β-lactam-sensitive *M. caseolyticus* strain KM1352 (Table 1). The *mec* gene was designated *mecD* according to the SCC*mec* guidelines for reporting new *mecA* gene homologues which defines a new *mec* gene type if the nucleotide (nt) sequence shares less than 70% identity to any known *mec* genes^13^. The *mecD* gene was identical in strains IMD0819, IMD0473 and KM0211 and shared 69% nt identity and 63% amino acid (aa) identity with *mecB* of *M. caseolyticus* strain JCSC5402^1^ and less than 62% nt and 53% aa identity with all other PBP2a of the Mec family (Fig. 1).

### Regulation and expression of *mecD*

#### The *mecD* gene complex

The *mecD* gene was preceded by two regulatory genes *mecR1*_*m*_ and *mecI*_*m*_ transcribed in the opposite direction. The gene organization was similar to regulated systems containing other *mec* structural genes or *blaZ*. Comparison of *mecD* regulatory genes with these systems demonstrated that they possessed the highest sequence similarity with those of *mecB*, namely 61% nt identity with the homologous sensor/transducer gene *mecR1*_*m*_ and 70% with the transcriptional repressor gene *mecI*_*m*_ of *M. caseolyticus* JCSC5402 (Supplementary Figs S1 and S2). The genomes of the *mecD*-containing *M. caseolyticus* strains contained no further *mec/bla* regulators and no homologous genes to the β-lactamase *blaZ* or the antirepressor *mecR2*. The absence of a β-lactamase was confirmed by negative results in the nitrocefin test.

#### Promoter-operator structure

**Figure 2:**
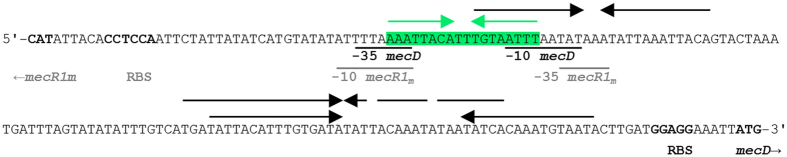
Divergent promoters and operator sequence in the intergenic region between *mecR1*_*m*_ and *mecD* genes of *M. caseolyticus*. The −10 and −35 promoter sequences are underlined. Start codons and ribosomal binding sites (RBS) are in bold type. The operator sequence is highlighted in green and inverted repeats marked by arrows. The consensus sequence [A/G]NATTACA[A/T]NTGTA [A/G][T/G]NT (with bases acceptable for one given position between square brackets and “N” for any base) was used to identity operator sequence recognized by MecI/BlaI repressors^27^. The sequence is shown for *M. caseolyticus* IMD0819 (Genbank acc. no KY013611, position 15264–15440).

Analysis of the 171-bp intergenic region between *mecD* and *mecR1*_*m*_*-mecI*_*m*_ revealed a divergent promoter pair for bidirectional transcription in an overlapping 32-bp fragment (Fig. 2). Promoters were predicted using the program BPROM that recognizes −35 and −10 consensus sequences for the bacterial sigma factor 70^26^. In addition, an operator site was identified using the consensus sequence for *mec/bla* divergons defined by Garcia-Castellanos^27^ (Fig. 2). The operator site is located within the *mecR1*_*m*_ and *mecD* promoter region suggesting that binding of one MecIm dimer can repress transcription of *mecD* and *mecR1*_*m*_*-mecI*_*m*_ simultaneously. The perfect consensus sequence for the *mec/bla* divergon found in *M. caseolyticus* suggests possible control of *mecD* expression by other MecI/BlaI proteins as is the case in *S. aureus* where BlaI and MecI can exchange with each other^20^,^28^. In addition to the palindromic sequences present in the *mec* operator, perfect and imperfect inverted repeats were found in the region between the *mecD* promoter and start site that might function as regulatory sequence in genomic DNA or RNA transcript (Fig. 2).

#### *mecD* regulators: MecR1m and MecIm

Resistance to β-lactams mediated by *blaZ* and *mecA* is controlled through an inducible proteolytic signal transduction pathway in *S. aureus*^18^,^29^. The *mecD* regulators were therefore analyzed for the presence of aa-patterns and domains known to play a functional role in this pathway. MecR1m contained a putative C-terminal penicillin-binding domain (PBD) involved in β-lactam sensing (Ser315-Lys561). The three signature motifs conserved within PBPs/β-lactamases were also present^30^: motif 1 including the catalytic serine (SxxK) was found at aa position 369–372 (Ser-Thr-Tyr-Lys), motif 2 (SxN) at position 417–419 (Ser-Val-Asn) and motif 3 (KTG[T/S]) at position 504–507 (Lys-Thr-Gly-Thr). The protein also showed a classical zinc-binding motif (HExxH) at position 183–187 (His-Glu-Ile-Thr-His) and a potential autolytic cleavage site (Lys272-Arg273) for activation of the metalloproteinase according to BlaR^18^.

The MecIm repressor was predicted to contain an N-terminal DNA-binding domain (Met1-Val73) with a winged helix-turn-helix structure matching entries cl21459 and pfam12802 in the Conserved Domain Database (CDD) (https://www.ncbi.nlm.nih.gov/cdd/). The putative recognition helix (Ser41-Asn56) was the most conserved segment that shared 80% sequence identity with the recognition helix α3 of the MecI protein of *S. aureus* N315^27^, while the overall aa identity was much lower with only 47% identity (Supplementary Fig. S2). The potential cleavage site for MecIm repressor inactivation was found between Asn101-Phe102 corresponding to the sites demonstrated in staphylococcal MecI and BlaI^18^,^29^.

#### MecD

The *mecD* gene encodes a 678-aa protein with a C-terminal transpeptidase domain (Ser330-Glu678) (CDD: pfam00905 and cl21491) that contains the three signature motifs conserved within PBPs (see paragraph *mecD* regulators). Motif 1 was present at position 406–409 (Ser-Thr-Gln-Lys), motif 2 at position 465–467 (Ser-Asp-Asn) and motif 3 at position 605–608 (Lys-Thr-Gly-Thr) followed by an alanine typically found in class B PBPs. The N-terminal section consisted of a transmembrane-helix (Lys7-Leu25, N-terminus inside) as predicted using TMpred software (http://www.ch.embnet.org/) and a non-penicillin binding domain (nPBD) (Glu27-Leu329) with an N-terminal extension subdomain (Glu27-Ala140) (CDD: pfam05223) typically found in PBP2a. Compared to MecA, MecD showed a distantly related nPBD and a more conserved transpeptidase domain with 37% and 64% aa identity, respectively.

### **mecD*-mediated resistance phenotype*

Minimal inhibitory concentrations (MICs) of different classes of β-lactam antibiotics were determined for the three *mecD*-positive *M. caseolyticus* strains and the *mecD*-negative strains KM1352 and CCUG 15606 T (Table 2). As control, two *mecD*-deletion mutants of strain IMD0819 were included. These mutants had lost either an 18,134-bp (strain IMD0819_20) or a 20,907-bp (strain IMD0819_33) genomic element containing the *mecD* gene (see paragraph construction of McRI_mecD_-1 deletion variants). Compared to the *mecD*-negative strains, the *mecD*-containing strains IMD0819, IMD0473 and KM0211 showed several fold higher MICs of penicillins including the penicillinase-resistant drugs oxacillin and temocillin. The MICs were also higher for 2^nd^ (cefoxitin), 3^rd^ (cefotaxime and ceftazidime) and 4^th^ (cefepime) generation cephalosporins as well as for carbapenems (ertapenem, imipenem and meropenem). The MIC for the anti-MRSA cephalosporins, ceftobiprole and ceftaroline were also at least 16-fold (ceftobiprole) and 8-fold (ceftaroline) higher in the *mecD*-containing strains. The transpeptidase domain of MecD provides similar residues to those identified to be important for ceftobiprole binding within the active site region of MecA (Tyr446, Thr600, Met641)^31^, namely Phe449, Thr608, Met650. The Tyr to Phe substitution in MecD should not decrease ceftobiprole binding since only aromatic stacking interaction was observed between Tyr446 and ceftobiprole^31^. The reason for reduced susceptibility may be associated with other structural differences between MecA and MecD, e. g. the diverse nPBDs. The nPBD (also called allosteric domain) of MecA is involved in allosteric control of the transpeptidase’s active site^32^. Ceftaroline binds to allosteric sites in the nPBD, causing a rearrangement of salt bridges and predisposes MecA to acylation by a second β-lactam molecule^32^. The nPBD of MecD is only distantly related to that of MecA and neither equivalent residues for ceftaroline binding nor a similar scatter of charged aa are obvious which questions an analogue allosteric control mechanism for MecD.

The complete structure of *mecR1*_*m*_-*mecI*_*m*_ suggests inducible *mecD* expression in the presence of β-lactams. To study *mecD* expression, two plasmids were constructed using *S. aureus*-*E. coli* shuttle vector pTSSCm^33^. Plasmid pTSSCm-D1 contained *mecD* with its upstream 171-bp intergenic region and plasmid pTSSCm-D2 contained the entire *mecD* operon including the regulator genes *mecR1*_*m*_ and *mecI*_*m*_. Both plasmids were electroporated into *S. aureus* RN4220 and selected for tetracycline resistance encoded on the vector. Transformants were only readily obtained with pTSSCm-D2 which contained the regulated *mecD* operon but not with pTSSCm-D1 which carried unregulated *mecD*. RN4220 colonies harboring pTSSCm-D1 were only obtained using Chromagar MRSA II for selection. These colonies grew slowly when re-streaked on agar containing tetracycline and only recovered their normal growth after several passages. They presented high level β-lactam resistance after the first subculturing but the level of resistance decreased after subsequent passages (e.g. MIC of oxacillin dropped from 32 mg/L to 2 mg/L). These results indicated that constitutive *mecD* expression has a deleterious effect and expression level needs to be downregulated for growth in the absence of β-lactams in *S. aureus*. Similarly, instability of unregulated plasmid-carried *mecA* was also observed in *S. aureus*, where the presence of *mec* or *bla* regulators was demonstrated to stabilize *mecA* integrity and expression^34^.

RN4220 cells carrying unregulated *mecD* on the plasmid pTSSCm-D1 presented MICs of β-lactams all above the values measured for RN4220 alone or those containing the empty vector (Table 2). Cells harboring pTSSCm-D2 with the regulated *mecD* showed 8-fold increase in the MIC of cefoxitin and at least 4-fold increased values for oxacillin, ceftaroline and cefotaxime, but no increase of MIC for ceftobiprole, ertapenem and imipenem indicating that these β-lactams are poor inducer for *mecD* expression in *S. aureus*. Importantly, in the presence of *mecD*, cefoxitin MIC values were always measured above the EUCAST breakpoint defined for MRSA screening (MIC: R > 4)^35^, also confirming the production of a low affinity PBP2a in RN4220. Compared to *S. aureus*, phenotypic resistance mediated by *mecD* was remarkably higher in *M. caseolyticus* with all the tested β-lactams (Table 2). Production of MecD seems to be well adapted for *M. caseolyticus* and to be suboptimal for *S. aureus*. Expression of an additional PBP may interfere with cell wall metabolism and needs to be regulated. In *S. aureus*, MecI functions as a strong transcriptional repressor and prompt induction and expression of *mecA* is usually not achieved if it is solely controlled through the *mecR1-mecI* regulators^19^,^20^. Enhanced expression of β-lactam resistance is observed for *mecA* controlled through the *blaZ* regulators and also the anti-repressor *mecR2*^19^,^20^,^21^,^36^. It might be possible that the *mecD* phenotype could be stronger in another *S. aureus* background than RN4220, that lacks both *mecR2* and *blaR1-blaI*.

### Characterization of chromosomal resistance islands carrying *mecD*

#### McRI_*mecD*_-1

**Figure 3:**
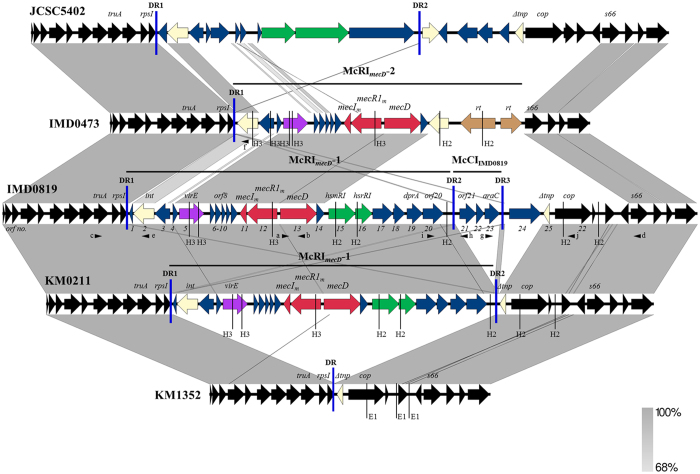
Structures of McRI_*mecD*_-1 and McRI_*mecD*_-2 and flanking sequences. Comparison was performed with sequences of *M. caseolyticus* strains JCSC5402 (Genbank acc. no. NC_011999, position 215180–250630), IMD0473 (Genbank acc. no KY013610), IMD0819 (Genbank acc. no KY013611), KM0211 (Genbank acc. no KY013612) and KM1352 (Genbank acc. no KY013613) using Easyfig software^57^. Gray areas indicate regions with between 68% to 100% nucleotide sequence identity. Regions encompassing McRI_*mecD*_-1, McRI_*mecD*_-2 and McCI_IMD0819_ are indicated by horizontal black lines and flanking direct repeats (DRs) by vertical blue lines. The open reading frames (*orfs*) are represented by arrows: *mecD, mecR1*_*m*_ and *mecI*_*m*_ are shown in red, *orfs* encoding integrase (*int*) or transposase (*tnp*) are shown in yellow, reverse transciptases (*rt*) in beige, *orfs* associated with restriction-modification in green and virulence-associated *orfs* in mauve; the *orfs* occurring in all *M. caseolyticus* strains are shown in black and additional strain-specific *orfs* in blue. The primers used in this study are indicated by small black arrowheads (**a**), mecD-F; (**b**), mecD-R; (**c**), truA-F; (**d**), s66-R; (**e**), int-0819-F; (**f**), int-0473-F; (**g**), araC-F; (**h**), orf21-R; (**i**), orf20-F; (**j**), cop-R) and cleavage sites for restriction endonucleases HindIII (H3) and HincII (H2) and EcoRI (E1) by thin vertical lines.

The *mecD* gene was located on a nearly identical 18,134-bp element (99.97% nt identity) in strains IMD0819 and KM0211. The element was designated *M. caseolyticus* resistance island *mecD* one (McRI_*mecD*_-1). In both strains, McRI_*mecD*_-1 was integrated into the 3′ end of the 30S ribosomal protein S9 gene (*rpsI*), flanked by imperfect extended direct repeats (DR1 and DR2) of either 160, 161 or 163 bp (Fig. 3). At the 5′ end, McRI_*mecD*_-1 carried an open reading frame (*orf*) encoding an integrase (*int*) of the tyrosine recombinase family and two upstream divergent oriented *orfs* coding for putative DNA-binding proteins with helix-turn-helix (HTH) motifs, which belong either to the transcriptional Cro/C1-type (*orf3*) or to the excisionase/Xis family (*orf4*). The gene organization resembles the *int*-*stl(*-*str*)-*xis* structure found in SaPIs of *S. aureus*, but further characteristics of these phage-related chromosomal island like *pri, rep, pif* and *terS* genes^37^ were absent in McRI_*mecD*_-1. Besides the *mec* operon, McRI_*mecD*_-1 contained 17 predicted *orfs*, including one gene encoding a potential virulence factor (*virE*), genes for restriction-modification system (*hsmRI* and *hsrRI*) and for a putative DNA recombination-mediator protein (*dprA*) (Fig. 3). VirE contained the virulence-associated protein E domain (CDD: pfam05272) and shared 36% aa identity with the VapE protein encoded on SePI_*fusB*-857_ of *S. epidermidis* NTUH-857^38^. The DNA modification methyltransferase HsmMI and the restriction endonuclease HsrRI displayed 46 and 65% aa identity to those enzymes of the BsuBI/PstI type II system of *B. subtilis*, respectively^39^. DprA of McRI_*mecD*_-1 was up to 51% identical to homologues of *Bacillus* species (WP_034289281) but only 30% identical to the native homologue of *M. caseolyticus* (GenBank: WP_012656740). DprA proteins are ubiquitously found in bacteria. In *Bacillus subtilis*, DprA has been shown to be involved in natural competence and mediating homologous recombination through recruitment of RecA to ssDNA^40^,^41^.

McRI_*mecD*_-1 represents a new element that shows only fragmentary sequence identity with GenBank entries. The fragment containing the *int* gene (position 7261–8482 in IMD0819, GenBank acc. no KY013611) shared 77% nt identity with a unique integrase in the chromosome of *M. caseolyticus* JCSC5402 (Fig. 3) (GenBank acc. no NC_011999) and in the draft genome of *S. aureus* 930918–3 (ABFA01000015.1). In addition, the genome of *M. caseolyticus* JCSC5402 and *S. aureus* 930918–3 shared 85 and 88% identity with a fragment containing the *orf8* of McRI_*mecD*_-1 (position 12182–12418 in IMD0819), respectively. The same fragment was also found with 81% identity in the draft genome of *S. hominis* LRKNS031 (LXRS01000085.1). The fragment containing the *mec* operon of McRI_*mecD*_-1 (position 13185–17475 in IMD0819) shared 66% overall identity with the *mecB*-containing fragment of the plasmid pMCCL2 of *M. caseolyticus* JCSC5402 (AP009486.1), indicating a novel *mec* operon type^13^ on the new element McRI_*mecD*_-1.

Downstream of McRI_*mecD*_-1, strain IMD0819 contained 4 additional *orfs (orf21-orf24*) and another DR (DR3) not present in the other *M. caseolyticus* strains (Fig. 3). The 404-bp DR3 shared 91% identity overall with a 405-bp region containing DR2. The segment between DR2 and DR3 represented a 2,773-bp chromosomal island (CI) with three *orfs* and was called McCI_IMD0819_. Together, McRI_*mecD*_-1 and McCI_IMD0819_ constituted a composite island in IMD0819. While only partial sequence similarity (coverage <20%) was found between McCI_IMD0819_ and *Staphylococcus/Bacillus* GenBank entries, McCI_IMD0819_ showed 97% overall identity to *E. faecium* strains UC7265 (JRHQ01000038.1) and UC7267 (ASAM01000028.1). The three *orfs (orf21-orf23*) of McCI_IMD0819_ encode hypothetical proteins: ORF21 contained a putative domain of bacteriocin-processing endopeptidases (CDD: cl00296) and ORF23 contained a domain for AraC family transcriptional regulators (CDD: COG3708). ORF24, situated downstream of McCI_IMD0819_, encoded a putative AAA family ATPase that shared 44% aa identity with a protein of a *Bacillus* species (WP_069304013).

#### McRI_*mecD*_-2

McRI_*mecD*_-2 consisted of a 16,188-bp insert in the chromosome of IMD0473. It was delimitated by the attachment (*att*) site defined at the 3′ end of *rpsI* (see paragraph excision and circularization of McRI_*mecD*_-1-McCI_IMD0819_ subunits) and the s66 family peptidase gene (*s66*) identified as a core genome sequence in all other *M. caseolyticus* strains (Fig. 3). A DR at the right side of McRI_*mecD*_-2 was missing as well as a chromosomal segment including the *cop* gene suggesting that deletion took place at this locus in IMD0473. The *mecD*-containing segment of McRI_*mecD*_-2 was similar to that of McRI_*mecD*_-1 (position 11280–17867 in IMD0819) comprising *orf6* to *orf14* (99.97% nucleotide identity) (Fig. 3). However, McRI_*mecD*_-2 contained different integrase and *virE* genes with 77% identity and 75% identity to those of McRI_*mecD*_-1 at the left side, and a completely different sequence on the right side, which carried a putative transposase and two possible reverse transcriptases (RTs). The transposase displayed similarity to those of the IS*30* family (CDD: COG2826), and was also present in the genome of IMD0819. It shared up to 51% aa identity with transposases found in *Staphylococcus* and *Enterococcus* species. The RTs displayed the RT motifs 3, 4 and 5 and a domain typically found in bacterial retrotransposons and retrons (CDD: cd01646)^42^. Both proteins showed less than 40% aa identity with all other GenBank entries.

The chromosomally integrated structures of McRI_*mecD*_-1 and McRI_*mecD*_-2 were confirmed by HindIII and HincII restriction analysis of long-range PCR products spanning *mecD* with the 5′ end region and 3′ end region of the islands, respectively (Fig. 3). The amplicons were obtained using primer pairs mecD-R and truA-F and mecD-F and s66-R (Fig. 3 and Supplementary Table S1). The structure of the *mecD*-negative strain KM1352 was verified by EcoRI digest of the PCR product amplified with primers truA-F and s66-R.

### Mobility of McRI_*mecD*_-1 and McRI_*mecD*_-2

#### Site-specific integrases associated with *mecD*

**Figure 4:**
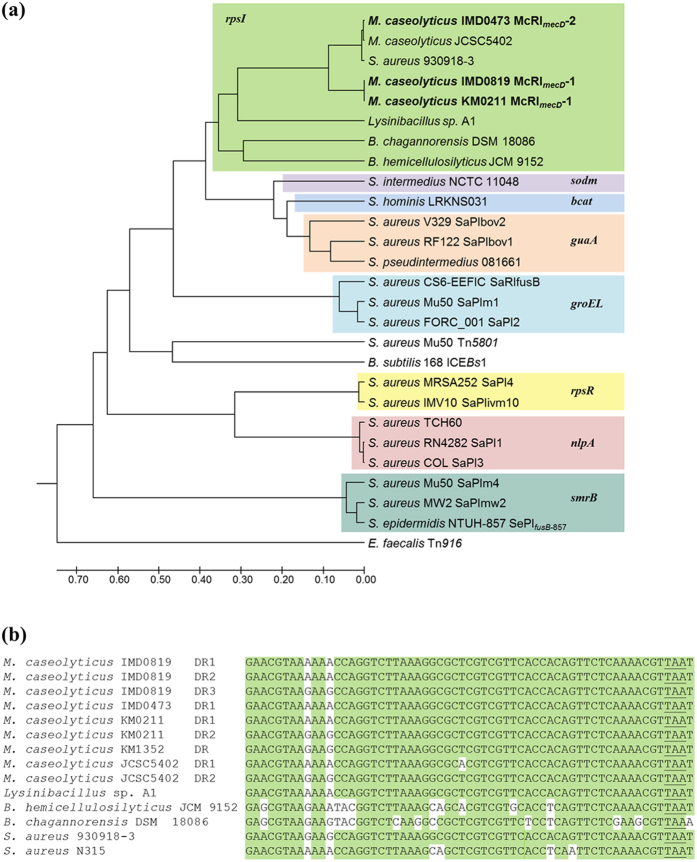
(**a**) Phylogenetic tree of integrases of the tyrosine recombinase family. Colored boxes group members that share homologous integration sites (*rpsI*, gene for 30S ribosomal protein S9; *sodm*, superoxide dismutase gene; *bcat*, gene for branched-chain amino acid aminotransferase; *guaA*, GMP synthetase gene; *groEL*, chaperonin gene; *rpsR*, gene for 30S ribosomal protein S18; *nlpA*, gene for component of ABC-type metal ion transport system; *smrB*, gene for SsrA-binding protein). Analysis was performed for amino acid (aa) sequences using the UPGMA method in MEGA7^56^. Host strains and genetic elements if known are indicated. References for the used sequences can be found in Supplementary Table S3. (**b**) Putative core attachment sites recognized by *rpsI*-associated integrases. For *M. caseolyticus* strains, the imperfect direct repeats (DR) carrying the *att* consensus sequence are indicated. For all other species the *att* site found at the 3′ end of the *rpsI* gene is given. The *rpsI* stop codon is underlined. Positions that hold variant bases are unshaded. All species, except *S. aureus* N315 and *M. caseolyticus* KM1352, carry an integrase gene downstream of *rpsI*.

The integrase situated on the 5′ end of the *mecD* resistance islands may catalyze integration and excision of the elements. The integrases in McRI_*mecD*_-1 and McRI_*mecD*_-2 differed slightly from each other, sharing 81% aa identity. Both consisted of a 388-aa protein that contained a N-terminal SAM-like domain found in phage integrases (CDD: pfam14569) and the conserved residues described for tyrosine recombinase in the C-terminal section, including the active site tyrosine at position 367 and two invariant arginines at position 210 and 334^43^. The integrase of McRI_*mecD*_-2 was 98% identical to the integrases found in the chromosome of *M. caseolyticus* JCSC5402 and in the draft genome of *S. aureus* 930918–3, both strains being negative for *mecD*. In JCSC5402, the integrase was associated with a unique sequence that encodes a putative type III restriction modification system and was also delimitated by DRs (Fig. 3). In *S. aureus* 930918–3 and the *M. caseolyticus* strains JCSC5402, IMD0819, KM0211 and IMD0473, the integrase was inserted downstream of the *rpsI* gene, suggesting a site-specific activity of the enzyme. Distantly related integrases that share up to 46% aa identity with the integrases of *M. caseolyticus* strains were also found next to the *rpsI* gene in *Bacillus* species (Fig. 4a). Compared with other integrases of the tyrosine recombinase family, *mecD*-associated integrases showed 40 to 43% identity to the integrases of SaPIbov1/2 and of uncharacterized inserts in *S. intermedius, S. hominis* and *S. pseudintermedius* (Fig. 4a). The analysis suggests that the integrases of *mecD* resistance islands have a common ancestor with *rpsI*-associated integrases of *Bacillus* and the potential to transfer genetic information to *S. aureus* and probably other staphylococci.

#### Excision and circularization of McRI_*mecD*_-1-McCI_IMD0819_ subunits

Spontaneous formation of circular DNA molecules containing *mecD* was tested by PCR and sequencing using divergent primers specific for *mecD* and *int* (primers a and e/f in Fig. 3). Two PCR products were obtained with IMD0819, one product with KM0211 and none with the McRI_*mecD*_-2 containing strain IMD0473 (Supplementary Table S4). PCR was also performed to detect the chromosomal segment remaining after excision of the McRI_*mecD*_-1 subunits using convergent primers specific for *truA* and *cop* (primers c and j in Fig. 3) and a short elongation time to avoid amplification of the entire insert. The resulting fragments indicated one deletion in KM0211 and two deletion variants in IMD0819 (Supplementary Table S4). Furthermore, PCR products for possible excision of McCI_IMD0819_ in strain IMD0819 were obtained using divergent (primers g and h) and convergent primer (primers i and j) pairs placed inside and outside of McCI_IMD0819_, respectively. Sequencing results confirmed circularization of McRI_*mecD*_-1, composite McRI_*mecD*_-1-McCI_IMD0819_ and McCI_IMD0819_ caused by recombination between DRs. Consistently, the left DR was incorporated in the circular molecule and the right DR remained as a joining region on the chromosome (Supplementary Table S4). This pattern suggests site-specific as well as orientation-specific enzymatic activity most probably encoded by the integrase of the *mecD* resistance islands. Homologous recombination mediated by DprA protein also encoded on McRI_*mecD*_-1 would lead to a random recombination of DRs. The absence of circular McRI_*mecD*_-2 molecule in IMD0473 can be explained by the lack of flanking DRs. A 61-bp core *att* site representing a putative target for the integrase was found in the extended imperfect DR sequences. This consensus sequence includes the 3′ end of *rpsI* gene and was also found in *S. aureus* and *Bacillus* species (Fig. 4b). In *M. caseolyticus, att*-DR1 of IMD0819, KM0211 and IMD0473 were identical and specified to be part of the *mecD* resistance islands. On the other hand, *att*-DR2 of KM0211 included the displaced 3′ end of *rpsI* after McRI_*mecD*_-1 insertion and was identical to the DR of the methicillin-sensitive strain KM1352. Notably, positions of mismatches clarified that cleavage for strand exchange must have taken place among the first 8 bases of the *att* sites (Fig. 4b).

##### Construction of McRI_*mecD*_-1 deletion variants

To induce excision of McRI_*mecD*_-1, cells were grown in the presence of subinhibitory concentrations of ciprofloxacin. This treatment has been reported to induce SOS response, excision of bacteriophages and the movement of SaPIs^44^,^45^. Using replica plating, two cefoxitin-susceptible clones were obtained from IMD0819 (2 of 251 clones tested) but none from KM0211 (0 of 445). Sequencing revealed excision of McRI_*mecD*_-1 in the deletion mutant IMD0819_20 and the composite element McRI_*mecD*_-1-McCI_IMD0819_ in the deletion mutant IMD0819_33. The chromosomal segments were joined by DR2 in IMD0819_20 and DR3 in IMD0819_33. Both strains had lost β-lactam resistance as confirmed by susceptibility measurement (Table 2).

## Conclusions

The novel *mecD* gene has been demonstrated to confer resistance to all classes of β-lactams including anti-MRSA cephalosporins, ceftobiprole and ceftaroline in *M. caseolyticus*. A transfer to *S. aureus* may jeopardise the efficacy of the last generation cephalosporins in MRSA. The *mecD* gene was located on genomic islands McRI_*mecD*_-1 and McRI-_*mecD*_-2 associated with a putative virulence gene and a site-specific integrase suggesting a potential for dissemination. Although a mechanism of horizontal gene transfer is not obvious due to the absence of genes for conjugative transfer or for interaction with phage packaging machinery, circular excisions containing *mecD* were observed. This characteristic resembles SCC*mec* elements that also do not encode genes for transfer but serine recombinases capable of element excision and circularization^23^,^46^. The integrase of the tyrosine recombinase family located on *mecD*-resistance islands is suggested to recognize a conserved core *att* site present at the 3′ end of *rpsI* gene. Conservation of this *att* site in *Bacillus* and *Staphylococcus* species and the detection of a similar *rpsI*-associated integrase in the *S. aureus* strain 930918–3^47^ suggest a potential for *mecD* elements to be acquired also by *Staphylococcus* species.

The presence of novel genetic elements containing a new methicillin- resistance gene in clinical *M. caseolyticus* strains from animal origin emphasizes once again the potential of bacteria to adapt to novel environments and to resist antimicrobial selective pressure of β-lactam antibiotics, which are widely used in veterinary medicine.

## Materials and Methods

### Bacterial strains and growth conditions

The origin and characteristics of the *M. caseolyticus* strains used in this study are listed in Table 1. They were obtained from the diagnostic unit of the Institute of Veterinary Bacteriology at the University of Bern. The samples were taken by veterinarians for diagnostic purposes therefore not requiring ethical approval or a permit for animal experimentation according to the current Swiss legislation (Federal Animal Protection Law, 455 (https://www.admin.ch/opc/de/classified-compilation/20022103/index.html). Strains were routinely cultivated on trypticase soy agar plates containing 5% sheep blood (TSA-SB) (Becton, Dickinson and company, Franklin Lakes, NJ, USA) at 37 °C. Species identification was performed using matrix-assisted laser desorption/ionization time-of flight mass spectrometry (MALDI-TOF MS) (microflex LT, Bruker Daltonics, Bremen, Germany). The laboratory strains *E. coli* DH5α and *S. aureus* RN4220^48^ were used for cloning and transformation experiments. They were cultivated in Luria-Bertani (LB) broth with shaking or on LB agar plates at 37 °C under aerobic conditions. The recombinant DH5α and RN4220 strains containing the *S. aureus-E. coli* shuttle vector pTSSCm^33^ or derived constructs were selected and routinely grown using 10 mg/l tetracycline in the growth medium.

### DNA preparation and PCRs

Plasmid DNA and genomic DNA were isolated using the peqGOLD Plasmid Miniprep Kit I and the peqGOLD Bacterial DNA kit (Peqlab Biotechnologie GmbH, Erlangen, Germany), respectively. To improve lysis of *M. caseolyticus*, cells were first incubated in Solution I of the kit supplemented with 50 mg/l of lysostaphin (Sigma-Aldrich, St Louis, MO, USA) and 2 g/l of lysozyme (Roche Diagnostics, Rotkreuz, Switzerland) for 20 min at 37 °C. For analytical PCR reactions, FIREPol^®^ DNA polymerase (Solis BioDyne, Tartu, Estonia) and GoTaq^®^ Long PCR Master Mix (Promega, Madison, WI, USA) were used for short (<2.5 kb) and long amplicons (up to 20 kb), respectively. Insert amplifications for plasmid construction were performed using High-Fidelity DNA polymerases (Pfu DNA polymerase [Promega] or the Phusion Hot Start II High-Fidelity DNA polymerase [Thermo Fisher Scientific, Waltham, MA, USA]) according to the manufacturer’s instructions. All relevant primers used in this study are listed in Supplementary Table S1. The presence of *mecD* was confirmed by PCR using primers mecD-F (5′-TCCTTTAGCGATAGATGGTGAA) and mecD-R (5′-CTCCCATCTTTTCTCCATCCT).

### Genome sequencing and analysis

*M. caseolyticus* strains KM1352, IMD0819, IMD0473 and KM0211 were sequenced using Illumina MiSeq technology. Genomic DNA was extracted using the UltraClean^®^ Microbial DNA Isolation Kit (MO BIO Laboratories, Carlsbad, CA, USA). Library preparation and sequencing were performed according to the manufacturer’s standard protocols using MiSeq Reagent kit v2 (Illumina, Little Chesterfield, UK) at the Labormedizinisches Zentrum Dr. Risch, Bern-Liebefeld, Switzerland. Draft genomes were assembled *de novo* using Geneious version R9.1.5 (Biomatters, Auckland, New Zealand). Contigs were analyzed for the presence of antimicrobial resistance genes using BLAST and ResFinder^49^. To obtain larger scaffolds of region of interest (required for genome of IMD0819 and IMD0473), target contigs were aligned to the chromosome of *M. caseolyticus* JCSC5402 (GenBank: NC_011999) to identify adjacent contigs and gaps were filled by PCRs and Sanger sequencing (ABI PRISM 3100 genetic analyzer, Applied Biosystems, Foster City, CA, USA) (IMD0819: connection of contig1-contig21; IMD0473: connection of contig26-contig56-contig11). The genomic structure was subsequently confirmed by long-range PCR amplification and restriction analysis in all sequenced strains (Supplementary Table S2). Prodigal software for gene finding in prokaryotes was used to define *orfs*^50^. Annotation of the *orfs* was performed manually by BLAST homology and putative function of the translated *orfs* analyzed against Prosite entries^51^ and Conserved Domain Database (CDD)^52^. Spontaneous formation of circular DNA molecules and the chromosomal region remaining after excision was analyzed by PCR using specific divergent and convergent primer pairs and GoTaq^®^ Long PCR Master Mix (Supplementary Table S4). If more than one PCR product was obtained, fragments were gel purified prior to Sanger sequencing.

### Recombinant strains

Curing of *mecD* from *M. caseolyticus* IMD0819 was carried out using cells growing with shaking in LB broth and in the presence of subinhibitory concentrations of ciprofloxacin (0.1 or 0.025 mg/l) for 6 h at 37 °C. Dilutions were plated on TSA-SB and single colonies analyzed by replica plating using LB agar containing 5 mg/l cefoxitin for negative selection. Deletion of *mecD*-containing fragments in the susceptible clones was determined by PCR and Sanger sequencing.

Two recombinant plasmids for *mecD* expression were generated in *E. coli* DH5α and electroporated into *S. aureus* RN4220^53^,^54^. Insert sequences were obtained from *M. caseolyticus* strain IMD0819 through PCR amplification and introduced into the vector pTSSCm. The *mecD* gene including its native promoter was amplified with primers mecD-XhoI-F and mecD-SpeI-R (Pfu polymerase) and the *mecD*-*mecR1*_*m*_-*mecI*_*m*_ fragment was obtained using primers mec-XhoI-F and mecD-SpeI-R (Phusion Hot Start II High-Fidelity DNA polymerase) (Supplementary Table S1). The cloning primers carried a SpeI or a XhoI site in the 5′-overhang to facilitate ligation of the PCR products into the pTSSCm vector after restriction with XhoI and SpeI endonucleases. The new constructs were named pTSSCm*-*D1 (*mecD*) and pTSSCm-D2 (*mecD-mecR1*_*m*_-*mecI*_*m*_). Their structures were verified based on restriction digestion patterns and Sanger sequencing.

### Antimicrobial susceptibility testing

MICs were determined in Müller-Hinton broth through the microdilution technique^16^ using Sensititre EUST and EUVSEC2 plates (Thermo Fisher Scientific). Additionally, MICs of oxacillin (OXA), cefoxitin (FOX), penicillin (PEN), ceftobiprole (CBP) and ceftaroline (CRL) were determined using serial two-fold dilutions ranging from 0.25 mg/l to 128 mg/l (for OXA, FOX and PEN) and 0.125 mg/l to 64 mg/l (for CBP and CRL). Stock solution of ceftobirole (BAL0009141, Batch: 08004R25F) (Basilea Pharmaceutica AG, Basel, Switzerland) was prepared as described^55^. The dephosphorylated active form of ceftaroline (U3, Batch: CI 148/09) (AstraZeneca, Cambridge, UK) was freshly dissolved in 0.1 M sodium phosphate pH 7.5 at 1 mg/ml prior use. The production of β-lactamase was tested on BBL™ DrySlide™ Nitrocefin (Becton, Dickinson and Company).

### Data Availability

The nucleotide sequences of *mecD* resistance islands and flanking regions have been deposited in the GenBank under the accession number KY013611 for *M. caseolyticus* IMD0819, KY013612 for *M. caseolyticus* KM0211, KY013610 for *M. caseolyticus* IMD0473 and KY013613 for *M. caseolyticus* KM1352.

## Additional Information

**How to cite this article:** Schwendener, S. *et al*. Novel methicillin resistance gene *mecD* in clinical *Macrococcus caseolyticus* strains from bovine and canine sources. *Sci. Rep.* **7**, 43797; doi: 10.1038/srep43797 (2017).

**Publisher's note:** Springer Nature remains neutral with regard to jurisdictional claims in published maps and institutional affiliations.

## Supplementary Material

Supplementary Material

## Figures and Tables

**Table 1:** Origin and phenotypic and genotypic characteristics of *M. caseolyticus* strains used in this study.

*M. caseolyticus* strain	Origin/region	Resistance phenotype	Resistance genes	Reference
CCUG 15606 T	Bovine milk	—	ND	2
KM1352	Healthy dog/Jura	—	—	This study
IMD0819	Bovine mastitis milk/Fribourg	PEN, FOX, OXA, TET, KAN, TMP, STR	*mecD, tet*(L), *ant(4*′)*-Ia, dfrK, str*	This study
IMD0473	Bovine mastitis milk/Bern	PEN, FOX, OXA, TET, KAN, STR	*mecD, ant(4*′)*-Ia, tet*(L), *str*	This study
KM0211	Dog, otitis/Bern	PEN, FOX, OXA, STR	*mecD, str*	This study

ND: not determined.

Abbreviation of antimicrobials: PEN, penicillin; FOX, cefoxitin; OXA, oxacillin; TET, tetracycline; KAN, kanamycin; TMP, trimethoprim.

Antibiotic resistance genes and functions: *mecD*, penicillin-binding protein 2a; *tet*(L), tetracycline efflux protein; *ant(4*′)*-Ia*, kanamycin nucleotidyltransferase; *dfrK*, dihydrofolate reductase; *str*, streptomycin nucleotidyltransferase.

**Table 2:** Antimicrobial susceptibility of *Macrococcus caseolyticus* and *Staphylococcus aureus* strains to β-lactams as determined by broth microdilution.

Strain/plasmid	Characteristics	*mec* genes	MIC of β-lactam antibiotics (mg/L)											
			PEN	OXA	TMC	FOX	CTX	CAZ	FEP	CBP	CRL	ETP	IPM	MEM
*M. caseolyticus*														
CCUG 15606 T	Type strain	—	≤0.25	≤0.25	32	1	0.5	4	0.5	≤0.125	≤0.125	0.25	≤0.12	0.06
KM1352	Field strain	—	≤0.25	0.5	64	2	1	8	1	≤0.125	0.25	0.5	≤0.12	0.12
IMD0819	Field strain	*mecD-*mecR1*_*m*_-mecI*_*m*_	8	128	>128	64	>64	>128	>32	2	1	>2	8	4
IMD0473	Field strain	*mecD-*mecR1*_*m*_-mecI*_*m*_	8	>128	>128	64	>64	>128	>32	4	2	>2	4	8
KM0211	Field strain	*mecD-*mecR1*_*m*_-mecI*_*m*_	16	>128	>128	128	>64	>128	>32	8	2	>2	>16	8
IMD0819_20	IMD0819 ΔmcRI_*mecD*_-1	—	≤0.25	≤0.25	32	1	0.5	4	0.5	≤0.125	≤0.125	0.25	≤0.12	0.12
IMD0819_33	IMD0819 ΔmcRI_*mecD*_-1-McCI_IMD0819_	—	≤0.25	≤0.25	32	1	0.5	4	0.5	≤0.125	≤0.125	0.25	≤0.12	0.12
*S. aureus*														
RN4220	Recipient strain	—	≤0.25	≤0.25	ND	2	ND	ND	ND	0.5	0.25	ND	ND	ND
RN4220/pTSSCm	RN4220 with *S. aureus*-*E. coli* shuttle vector pTSSCm	—	≤0.25	≤0.25	64	2	1	8	2	0.5	0.25	0.12	≤0.12	0.12
RN4220/pTSSCm-D1	RN4220 with *mecD* cloned into pTSSCm	*mecD*	2	2	>128	32	16	64	8	1	1	2	0.5	2
RN4220/pTSSCm-D2	RN4220 with *mecD, mecR1*_*m*_ and *mecI*_*m*_ cloned into pTSSCm	*mecD-mecR1*_*m*_-*mecI*_*m*_	0.5	1	>128	16	4	16	4	0.5	1	0.12	≤0.12	0.25

Abbreviations: PEN, benzylbenicillin; OXA, oxacillin; TMC, temocillin; FOX, cefoxitin; CTX, cefotaxime; CAZ, ceftazidime; FEP, cefepime; CBP, ceftobiprole; CRL, ceftaroline; ETP, ertapenem; IPM, imipenem; MEM, meropenem.
