# Supplementary material for: Novel methicillin resistance gene mecD in clinical Macrococcus caseolyticus strains from bovine and canine sources
Source: Sci Rep. 2017 Mar 8;7:43797. doi: 10.1038/srep43797 (PMC5341023; doi:10.1038/srep43797)
Supplement: Supplementary Material [file srep43797-s1.pdf]

**Novel methicillin resistance gene *mecD* in clinical *Macrococcus caseolyticus* strains from bovine and canine sources**

Sybille Schwendener, Kerstin Cotting, Vincent Perreten*

Institute of Veterinary Bacteriology, Vetsuisse Faculty, University of Bern, Bern, Switzerland

*Corresponding author. Mailing address: Institute of Veterinary Bacteriology, University of Bern, Länggassstrasse 122, CH-3012 Bern, Switzerland, Phone: +41 31 631 24 30. Fax: +41 31 631 26 34. E-mail: vincent.perreten@vetsuisse.unibe.ch.

## Supplementary Figures

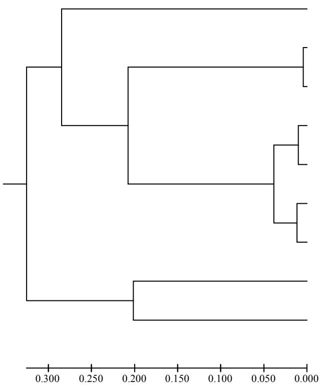


| | Identity (%) of | | | |
|---|---|---|---|---|
| **Gene** | **nt** | **aa** | **Strain** | **GenBank** |
| *blaR1* | 54 | 32 | *Staphylococcus aureus* NRS128 | NC_013319 |
| *mecR1* | 54 | 33 | *Staphylococcus fleurettii* SFMP08 | AB546267 |
| *mecR1* | 54 | 33 | *Staphylococcus aureus* N315 | NC_002745 |
| *mecR1* | 55 | 34 | *Staphylococcus stepanovicii* IMT28705 | KR732654 |
| *mecR1* | 55 | 34 | *Staphylococcus xylosus* S04009 | HE993884 |
| *mecR1* | 54 | 34 | *Staphylococcus sciuri* GVGS2 | HG515014 |
| *mecR1* | 53 | 33 | *Staphylococcus aureus* LGA251 | FR821779 |
| ***mecR1***$_m$ | **100** | **100** | ***Macrococcus caseolyticus* IMD0819** | **KY013611** |
| *mecR1*$_m$ | 61 | 49 | *Macrococcus caseolyticus* JCSC5402 | AP009486 |

Supplementary Figure S1. Phylogenetic tree of the receptors *mecR1*/*blaR1* of homologous systems consisting of *mec*-*mecR1*-*mecI* or *blaZ*-*blaR1*-*blaI* operon. Evolutionary analysis was performed for nucleotide sequences using the UPGMA method in MEGA7 [Mol. Biol. Evol. 2016; 33(7):1870-4.]. The percentage of nucleotide (nt) and amino acid (aa) identity between *mecR1*$_m$ of *M. caseolyticus* strain IMD0819 and other *mecR1*/*blaR1* receptors was determined by sequence alignment with Clustral OMEGA [http://www.ebi.ac.uk/Tools/msa/clustalo/]. Note: the *mecR1* sequence of *S. saprophyticus* strain 210 is only partially available in the GenBank and was therefore omitted. The *mecA1* gene of *S. sciuri* and the *mecA2* gene of *S. vitulinus* are not preceded by regulator genes.

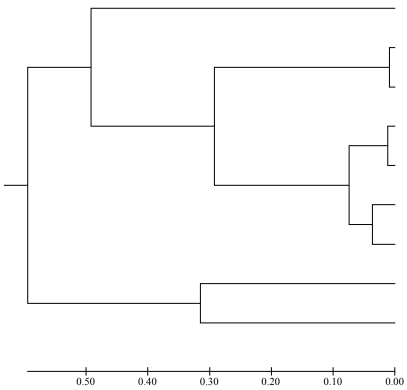


| | Identity (%) of | | | |
|---|---|---|---|---|
| **Gene** | **nt** | **aa** | **Strain** | **GenBank** |
| *blaI* | 58 | 41 | *Staphylococcus aureus* NRS128 | NC_013319 |
| *mecI* | 59 | 47 | *Staphylococcus fleurettii* SFMP08 | AB546267 |
| *mecI* | 59 | 47 | *Staphylococcus aureus* N315 | NC_002745 |
| *mecI* | 61 | 42 | *Staphylococcus stepanovicii* IMT28705 | KR732654 |
| *mecI* | 61 | 42 | *Staphylococcus xylosus* S04009 | HE993884 |
| *mecI* | 57 | 40 | *Staphylococcus sciuri* GVGS2 | HG515014 |
| *mecI* | 59 | 41 | *Staphylococcus aureus* LGA251 | FR821779 |
| ***mecI$_m$*** | **100** | **100** | ***Macrococcus caseolyticus* IMD0819** | **KY013611** |
| *mecI$_m$* | 70 | 61 | *Macrococcus caseolyticus* JCSC5402 | AP009486 |

Supplementary Figure S2. Phylogenetic tree of the transcriptional repressors *mecI*/*blaI* of homologous systems consisting of *mec*-*mecR1*-*mecI* or *blaZ*-*blaR1*-*blaI* operon. Evolutionary analysis was performed for nucleotide sequences using the UPGMA method in MEGA7 [Mol. Biol. Evol. 2016; 33(7):1870-4.]. The percentage of nucleotide (nt) and amino acid (aa) identity between *mecI$_m$* of *M. caseolyticus* strain IMD0819 and other *mecI*/*blaI* repressors was determined by sequence alignment with Clustral OMEGA [http://www.ebi.ac.uk/Tools/msa/clustalo/]. Note: *mecI* sequence of *S. saprophyticus* strain 210 is not available in the GenBank, and the *mecA1* gene of *S. sciuri* and the *mecA2* gene of *S. vitulinus* are not preceded by regulator genes.

## Supplementary Tables

Supplementary Table S1. Oligonucleotide primers.

| Primer name | Primer label in Figure 3 | Sequence (5'-3') | Target | Reference |
|---|---|---|---|---|
| mecA-1 | - | AAAATCGATGGTAAAGGTTGGC | *mecA* | J. Clin. Microbiol. 2002; 40(8):2786-90. |
| mecA-2 | - | AGTTCTGCAGTACCGGATTTGC | *mecA* | J. Clin. Microbiol. 2002; 40(8):2786-90. |
| mecB-fw | - | TGTTCGGCATTTCCGACGAA | *mecB* | Antimicrob. Agents Chemother. 2015; 59(8):4577-83. |
| mecB-rv | - | TCTCCCTGGCCATATCCTGA | *mecB* | Antimicrob. Agents Chemother. 2015; 59(8):4577-83. |
| mecC-F | - | CAGCCAGATTCATTTGTACC | *mecC* | J. Dairy Sci. 2013; 96(4):2247-57. |
| mecC-R | - | AACATCGTACGATGGGGTAC | *mecC* | J. Dairy Sci. 2013; 96(4):2247-57. |
| mecD-F | a | TCCTTTAGCGATAGATGGTGAA | *mecD* | This study |
| mecD-R | b | CTCCCATCTTTTCTCCATCCT | *mec*D | This study |
| mecD-XhoI-F | - | TTATA<u>CTCGAG</u>ATTACACCTCCAATTCTATTATATCATG | *mecD* operon | This study |
| mec-XhoI-F | - | TTATA<u>CTCGAG</u>CTGACTTCTCACTTAGTTTGAG | *mecD* operon | This study |
| mecD-SpeI-R | - | TTATA<u>ACTAGT</u>TTATTCTGAGTCTGGTTCATAAGTC | *mecD* operon | This study |
| truA-F | c | GACAGTATCCCTGCAATCATTC | *truA* | This study |
| s66-R | d | ACCAACCCGCATACTTCATC | *s66* | This study |
| int-0819-F | e | TGGCTAAGGACAAAGATCAG | *int* of McRI$_{mecD}$-1 | This study |
| int-0473-F | f | TCATGGCTTCAGGCATACAC | *int* of McRI$_{mecD}$-2 | This study |
| araC-F | g | TACCGTCATTCTGGCAAAC | *araC* | This study |
| orf21-R | h | GCTCCATTGCCCATAGAC | *orf21* | This study |
| orf20-F | i | GTATTCCCAACTTCGTCTGGA | *orf20* | This study |
| cop-R | j | GCAAGAATTAATACAATCCAATCTG | *cop* | This study |

Restriction sites used for cloning are underlined.

Supplementary Table S2. PCR and restriction analysis for detection of McRI$_{mecD}$-1 and McRI$_{mecD}$-2 in *M. caseolyticus*.

| Template | Forward primer (5′ region ) | Reverse primer (3′ region) | Amplicon size/bp | Restriction enzyme | Sizes of restriction fragment/bp |
|---|---|---|---|---|---|
| *mecD*-positive | | | | | |
| IMD0819 | truA-F | mecD-R | 11,030 | HindIII | 606 + 1,855 +3,854 + 4,715 |
| | mecD-F | s66-R | 19,771 | HincII | 994 + 1,621 + 2,551 +2,756 + 4,871 + 6,978 |
| KM0211 | truA-F | mecD-R | 11,030 | HindIII | 606 + 1,855 +3,854 + 4,715 |
| | mecD-F | s66-R | 14,920 | HincII | 994 + 1,621 + 2,127 + 2,551 + 2,756 + 4,871 |
| IMD0473 | truA-F | mecD-R | 10,846 | HindIII | 52 + 781 + 1,579 + 1,855 + 2,120 + 4,459 |
| | mecD-F | s66-R | 8,114 | HincII | 2,104 + 2,356 + 3,654 |
| *mecD*-negative | | | | | |
| KM1352 | truA-F | s66-R | 6,950 | EcoR1 | 607 + 1,346 + 1,584 + 3,413 |

Supplementary Table S3. References for integrases used to construct phylogenetic tree of Figure 4.

| Strain | Protein-ID | GenBank/ Nucleotide position | Reference |
|---|---|---|---|
| *M. caseolyticus* IMD0819 | | KY013611/ 8427-7261 | This study |
| *M. caseolyticus* KM0211 | | KY013612/ 8427-7261 | This study |
| *M. caseolyticus* IMD0473 | | KY013610/ 8233-7076 | This study |
| *M. caseolyticus* JCSC5402 | WP_012656138.1 | AP009484.1/ 223907-222741 | J. Bacteriol. 2009; 191(4):1180-90. |
| *S. aureus* 930918-3 | WP_000161346.1 | ABFA01000015.1/ 9951-11117 | BMC Genomics. 2008; 9:433. |
| *Lysinibacillus* sp. A1 | KHK50488.1 | JSZM01000021.1/ 109128-107962 | Genome Announc. 2015; 3(2). pii: e00095-15. |
| *B. chagannorensis* DSM 18086 | WP_026696990.1 | AUCK01000006.1/ 56493-55324 | Int. J. Syst. Evol. Microbiol. 2007; 57(Pt 9):2084-8. |
| *B. hemicellulosilyticus* JCM 9152 | GAE31526.1 | BAZO01000020.1/ 59332-58589 | Genome Announc. 2014; 2(1):e01258-13. |
| *S. intermedius* NCTC 11048 | WP_019168674.1 | CAIB01000160.1/ 37188-36052 | Front. Cell. Infect. Microbiol. 2012; 2:44. |
| *S. hominis* LRKNS031 | OAW30112.1 | LXRS01000041.1/ 43073-41937 | Direct Submission: Maurer, Baesler and Knobloch, 2016. |
| *S. aureus* V329 | AAP55251.1 | AY220730.1/ 25643-26779 | Mol. Microbiol. 2003; 49(1):193-210. |
| *S. aureus* RF122 | WP_000179355.1 | NC_007622.1/ 2037180-2038352 | PLoS One. 2007; 2(10):e1120. |
| *S. pseudintermedius* 081661 | WP_037541925.1 | CP016073.1/ 2667235-2668371 | Direct Submission: Riley, Bemis and Kania, 2016. |
| *S. aureus* CS6-EEFIC (SaRIfusB) | CAL23817.1 | AM292600.1/ 2580-1405 | J. Clin. Microbiol. 2007; 45(5):1505-10. Epub 2007 Mar 7. |
| *S. aureus* Mu50 (SaPIm1) | WP_000179343.1 | NC_002758.2/ 2147530-2148702 | Lancet. 2001; 357(9264):1225-40. |
| *S. aureus* Mu50 (Tn*5801*) | BAB56554.1 | NC_002758.2/ 437264-436071 | Lancet. 2001; 357(9264):1225-40. |
| *S. aureus* Mu50 (SaPIm4) | WP_000121223.1 | NC_002758.2/ 869568-868462 | Lancet. 2001; 357(9264):1225-40. |
| *S. aureus* FORC_001 | WP_000179345.1 | NZ_CP009554.1/ 2161377-2162549 | Microbiology. 2007; 153(Pt 10):3235-45. |
| *B. subtilis* 168 | NP_388361.1 | NC_000964.3/ 530611-529505 | |
| *S. aureus* MRSA252 | WP_000270135.1 | NC_002952.2/ 411390-410176 | Proc. Natl. Acad. Sci. U. S. A. 2004; 101(26):9786-91. |
| *S. aureus* IMV10 | BAM66883.1 | AB716349.1/ 2727-1510 | Microbiol. Immunol. 2013; 57(2):91-9. |
| *S. aureus* TCH60 | ADQ77825.1 | CP002110.1/ 2475780-2477024 | Direct Submission: Muzny et al., 2010 |
| S. *aureus* RN4282 | AAC28969.1 | U93688.2/ 13875-15095 | Mol. Microbiol. 1998; 29(2):527-43. |
| *S. aureus* COL | AAL04148.1 | AF410775.1/ 14574-15794 | J. Biol. Chem. 2002; 277(15):13138-47. |
| *S. aureus* MW2 | WP_025174195.1 | NC_003923.1/ 840811-839705 | Lancet. 2002; 359(9320):1819-27. |
| *S. epidermidis* NTUH-857 | BAO09125.1" | AB828060.1/ 2254-3911 | Antimicrob. Agents Chemother. 2013; 57(11):5737-9. |
| *E. faecalis* (Tn*916*) | AAB60030.1 | U09422/ 16641-17858 | Plasmid. 1994; 32(3):350-4. |

Supplementary Table S4. Excision of circular DNA intermediates from *M. caseolyticus* chromosome.

| Template | PCR product | PCR primers | Elongation time | Amplicon size/bp | Recombination sites | DR present in amplicon |
|---|---|---|---|---|---|---|
| | Circular intermediate | | | | | |
| IMD0819 | McRI$_{mecD}$-1 | int-0819-F and mecD-F | 15 min | 9,970 | DR1 and DR2 | DR1 |
| IMD0819 | McRI$_{mecD}$-1-McCI$_{IMD0819}$ | int-0819-F and mecD-F | 15 min | 12,563 | DR1 and DR3 | DR1 |
| IMD0819 | McCI$_{IMD0819}$ | orf21-R and araC-F | 1 min | 763 | DR2 and DR3 | DR2 |
| KM0211 | McRI$_{mecD}$-1 | int-0819-F and mecD-F | 15 min | 9,970 | DR1 and DR2 | DR1 |
| IMD0473 | none | int-0473-F and mecD-F | 7 min | - | - | - |
| | Chromosomal segment after excision | | | | | |
| IMD0819 | ΔMcRI$_{mecD}$-1 | truA-F and cop-R | 7 min | 7,075 | DR1 and DR2 | DR2 |
| IMD0819 | ΔMcRI$_{mecD}$-1-ΔMcCI$_{IMD0819}$ | truA-F and cop-R | 7 min | 4,301 | DR1 and DR3 | DR3 |
| IMD0819 | ΔMcCI$_{IMD0819}$ | orf20-F and cop-R | 2 min | 4,158 | DR2 and DR3 | DR3 |
| KM0211 | ΔMcRI$_{mecD}$-1 | truA-F and cop-R | 1.5 min | 2,224 | DR1 and DR2 | DR2 |
